# Salvia-Nelumbinis Naturalis Formula Improved Inflammation in LPS Stressed Macrophages via Upregulating MicroRNA-152

**DOI:** 10.1155/2017/5842747

**Published:** 2017-01-12

**Authors:** Zansong Ma, Xiangbing Shu, Jie Huang, Haiyan Zhang, Zhen Xiao, Li Zhang

**Affiliations:** ^1^Institute of Digestive Diseases, China-Canada Center of Research for Digestive Diseases (ccCRDD), Longhua Hospital, Shanghai University of Traditional Chinese Medicine, Shanghai 200032, China; ^2^The Fifth People's Hospital of Shanghai, Fudan University, Shanghai 200032, China

## Abstract

Salvia-Nelumbinis naturalis (SNN) formula is an effective agent in treating nonalcoholic steatohepatitis (NASH); however, the precise mechanisms are still undefined. Activation of Kupffer cells by gut-derived lipopolysaccharide (LPS) plays a central role in the pathogenesis of NASH. In the present study, we aimed to explore the epigenetic regulation of microRNAs under the beneficial effects of SNN-containing serum in LPS stressed macrophages. Kupffer cells were isolated from C57BL/6 mice and treated with LPS or LPS and SNN-containing serum; the mRNA expression of tumor necrosis factor-*α* (TNF-*α*) and interleukin-6 (IL-6) was assessed. By using microarray chips, we investigated differentially expressed microRNA profiles to decipher the underlining mechanisms of SNN-containing serum. It was revealed that SNN-containing serum decreased TNF-*α* and IL-6 expression, and microRNA-152 was identified as the potential epigenetic regulator. We further verified the pharmacological effects in Raw264.7 cells; while transfection with miRNA-152 mimics could reduce TNF-*α* and IL-6, transfection with miRNA-152 inhibitor blocked the anti-inflammatory effect of SNN-containing serum. These results suggested that SNN-containing serum could improve inflammation in LPS stressed Kupffer cells and macrophages via upregulating microRNA-152.

## 1. Introduction

Kupffer cells are specific liver macrophages with diverse functions in tissue homeostasis and disease onset, progression, and resolution. Resident Kupffer cells are originated from embryonic progenitor cells and seed in liver sinusoids and are in close contact with the liver sinusoidal endothelial cells [1, 2]. Kupffer cells function as guardians, scanning gut-derived microbial metabolites, and initial immunogenic responders against pathogenic compounds [3]. During inflammation and liver injury, they are assisted by infiltrating bone marrow-derived macrophages that originate from circulating monocytes.

At present, chronic liver disease frequently develops on the background of obesity, insulin resistance, dyslipidemia, and metabolic syndrome, with nonalcoholic fatty liver disease (NAFLD) being the most common cause [4]. NAFLD is defined as the presence of hepatic steatosis, which encompasses a spectrum ranging from simple steatosis to nonalcoholic steatohepatitis (NASH), fibrosis/cirrhosis, and even cancerous degeneration [5]. While simple steatosis is often considered to be benign, the presence of NASH indicated liver injury. According to the epidemiology data, estimated 20–25% NAFL patients will progress to NASH [6]; although the reason why the progression occurs in proportion is still unknown, studies implicate that Kupffer cells play crucial role in the spectrum that covers the pathogenesis of NAFLD-NASH and hepatocellular carcinoma (HCC) development [7]. Moreover, human NASH and independent HCC are characterized by an accumulation of macrophages around affected areas [8].

Currently, the need for specific pharmacotherapy in NASH treatment is urgent, yet the options available are limited. Natural products or herbs derived compounds provide important choices for treating NASH; however, while the effects can be evaluated, the underlining mechanisms are awaiting for exploration. Our group has designed Salvia-Nelumbinis naturalis (SNN) formula, initially called Jiangzhi Granula and specifically targeting NAFLD based on Traditional Chinese Medicine theories. The beneficial effects of SNN on NAFLD have been confirmed both in clinical trials and animal studies [9, 10]. Using methionine/choline deficient (MCD) diet-induced NASH mouse model, we have found that the SNN extracts could protect the liver from server damage [11]. Although we found antioxidant ability is one of the possible reasons [11], the exact mechanisms underlining the beneficial effect of SNN still need to be clarified.

MicroRNAs (miRNAs) are small noncoding RNAs that have generated much interest over the past decade. It functions in RNA silencing and posttranscriptional regulation of gene expression. miRNAs are well conserved and estimate to target about 60% of the genes of humans and other mammals. Innate immune responses and inflammation are fine-tuned by miRNAs [12]. Expression profiling studies have identified that tissue expression of miRNA can be differentially regulated in human liver diseases and in diverse pathophysiological settings that affect the liver [13]. miRNAs have been postulated to play a role in the pathogenesis of NASH [14], and the miRNAs profiles could indicate the specific pathways and targeted genes; thus screening miRNAs provides an efficient method for diagnosis and also for revealing potential mechanisms of certain drugs or compounds.

Considering the potential regulation of miRNAs in LPS induced TNF-*α* production and the importance of Kupffer cells activation in NASH, we specifically studied the role of SNN as an integrate agent, on Kupffer cells applying seropharmacological method. By screening the miRNA profiles, we looked into the mechanisms underlying the anti-inflammatory capacity of SNN-containing serum.

## 2. Materials and Methods

### 2.1. Experimental Animals

Male C57/BL6 mice (6–8 weeks, weighing 20 ± 0.5 g) and Wistar rats (8–10 weeks, weighing 270 ± 30 g) were purchased from SLAC Animal Laboratories (Shanghai, China). The mice were fed a standard rodent diet and water ad libitum. They were kept on a 12 h light/dark cycle in an animal facility. The animal protocols were performed in accordance with the guidelines and approval of the Animal Experiment Ethics Committee at Shanghai University of Traditional Chinese Medicine.

### 2.2. Isolation of Kupffer Cells from Mouse Liver

Mouse Kupffer cells were isolated by collagenase digestion and differential centrifugation, using Percoll as described previously [15]. Briefly, after the mouse was anesthetized with sodium pentobarbital, the abdomen was opened and the portal vein was cannulated with 16 G catheter. The liver was perfused, chopped, and suspended in collagenase solution supplemented with DNase. After 30 min digestion at 37°C, undigested portions were removed through cell strainer. The cell suspension was centrifuged, and the pellet was resuspended in serum-free RPMI 1640 medium, layered on a density cushion of 25%/50% discontinuous Percoll and then centrifuged. The cells floating at the boundary of the 2 Percoll layers were collected and washed in HBSS. Finally, the cells were suspended in RPMI 1640 medium containing 20% fetal bovine serum (FBS) and 1% antibiotic-antimycotic solution and plated in culture dishes (Falcon, Becton Dickinson, USA). After incubation for 4 h at 37°C with 5% CO2, nonadherent cells were removed by gently washing. The cells adhering to the dish were observed. The concentration of the cells was adjusted to 1 × 10^5^ cells/ml for the following experiment.

### 2.3. Kupffer Cells Identification

The purity of Kupffer cells was determined by F4/80 staining; cells were fixed in chamber slides with acetone-methanol (1 : 1) for 5 min at −20°C, washed 3 times with phosphate-buffered saline (PBS), and blocked with PBS containing 10% FBS for 30 min at room temperature before incubation with anti-mouse F4/80 antibody (1 : 100 dilution, overnight at 4°C, Abcam, USA). Subsequently, the cells were washed with PBS and incubated with FITC-conjugated anti-rat IgG (1 : 100 dilution, 1 h, room temperature, Sigma, USA). For the phagocytosis assay, Kupffer cells were incubated with ink containing medium for 2 h. Subsequently, the medium was removed, and the adherent cells were washed twice with PBS. The cells were observed under a fluorescence microscope. All photographs were taken with a digital camera (Olympus IX71).

### 2.4. SNN-Containing Serum Preparation and Identification

Male Wistar rats were randomly arranged in 2 groups (5 rats/group); rats of SNN group were gavaged SNN (composed of* Salviae*,* Nelumbinis*,* Rhizoma Polygoni Cuspidati,* and* Herba Artemisiae Scopariae*) in dose of 5.4 g/kg daily, while rats of vehicle group were gavaged the volume-matched saline. The treatment lasted for 7 days; on day 8, after 12 h of fasting, rats were subject to last administration, and at 4 h after administration, blood was retrieved from rats via aorta abdominalis with anesthesia by intraperitoneally injection of pentobarbital sodium at a dose of 40 mg/kg. Then, serum was collected by centrifugation of blood at 2000 ×g for 10 min at 4°C and heat-inactivated at 56°C for 30 min. All serum was sterilized by filtration through 0.22 *μ*m cellulose ester membranes. Some of the serum was qualified by High Performance Liquid Chromatography (HPLC), and the main ingredients it contained were identified.

### 2.5. Cell Viability Assay

Cell viability was determined by CCK-8 kit (Dojindo, Japan); the cells were plated on a 96-well plate and preincubated for 24 h. Then the cells were treated with or without the indicated concentrations of SNN-containing serum (5%, 10%, 15%) for 4 h; WST-8 solution was added to a final concentration of 10% (v/v). The converted orange products were measured in a microplate reader at 450 nm.

### 2.6. Cell Culture and Treatment

The isolated Kupffer cells and Raw264.7 cells were cultured in RPMI 1640 medium containing 20% FBS and 1% antibiotic-antimycotic solution. After a 72 h recovery/from isolation, the primary Kupffer cells were cultured in the absence or presence of 0.1 *μ*g/ml of LPS (Sigma, St. Louis, MO) for 12 h; then the LPS stressed cells were further added with different concentrations of SNN-containing serum (5%, 10%, and 15%) or saline serum for another 12 h.

### 2.7. Cell Transfection

The Raw264.7 cells grew in complete culture medium and were seeded in 96-well plate at the concentration of 10^4^ cells/well. The miRNA-152-3p mimics, inhibitors, and corresponding normal control (NC) were purchased from Tuoran Technologies (Shanghai, China). Lipofectamine 2000 and microRNA-152-3p inhibitor or mimics were prepared in sterile tubes. When the cells grew to 80% density, 50 nM miRNA-152-3p inhibitor and miRNA-152-3p mimics were transfected to the cells, respectively, and set inhibitor and mimics NC as the negative control. 4 h after the transfection, the medium was changed with fresh complete culture medium, and LPS was added with or without SNN-containing serum (10%) for another 12 h or 24 h. TNF-*α* and IL-6 levels in the medium were detected by ELISA kits (R&D systems) according to the manufacturer's instructions, and the cells were collected for mRNA detection.

### 2.8. Microarray Chip Analysis

Small RNAs were isolated from the total RNA of conventionally cultured Kupffer cells, LPS stressed Kupffer cells, and 10% SNN-containing serum treated Kupffer cells (LPS stressed). The Oebiotech Company performed the miRNA microarray assay. The fragmentation mixtures were hybridized to an Agilent 2100 (Agilent Technologies). Sample labeling, microarray hybridization, and washing were performed based on the manufacturer's standard protocols. Briefly, total RNAs were tailed with Poly A and then labeled with Biotin. After that, the labeled RNAs were hybridized onto the microarray. Having washed and staining the slides, the arrays were scanned by the Affymetrix Scanner 3000 (Affymetrix).

Affymetrix GeneChip Command Console software (version 4.0, Affymetrix) was used to analyze array images to get raw data and then offered RNA normalization. Next, Genespring software (version 13.1; Agilent Technologies) was used to proceed the following data analysis. Differentially expressed miRNAs were then identified through fold change as well as *P* value calculated using *t*-test.

### 2.9. Quantitate RT-PCR

Total RNA extracted by Trizol (Invitrogen, USA) was used to be reverse-transcripted into cDNA using AMV Reverse Transcriptase Kit (Promega, USA). Sequences of the primers (obtained from Shine Gene, Shanghai, China) used in the experiments were shown in Table 1. Quantitative real-time PCR (qRT-PCR) was performed using the SYBR Green PCR Master Mix kit (TOYOBO, Osaka, Japan) according to the manufacturer's protocol. Real-time PCR was performed on AB StepOne Plus PCR System (Applied Biosystems, Carlsbad, CA, USA). The relative mRNA levels were normalized using GAPDH as an internal control and expressed as fold change relative to the control.

### 2.10. Statistical Analysis

All the data was analyzed using GraphPad Prism 5.01 software (GraphPad Prism Software Inc., San Diego, CA) and presented as mean ± standard deviation (SD). Student's *t*-test was applied to assess data between different groups and *P* < 0.05 was considered statistically significant.

## 3. Results 

### 3.1. Kupffer Cells Isolation and Identification

**Figure 1:**
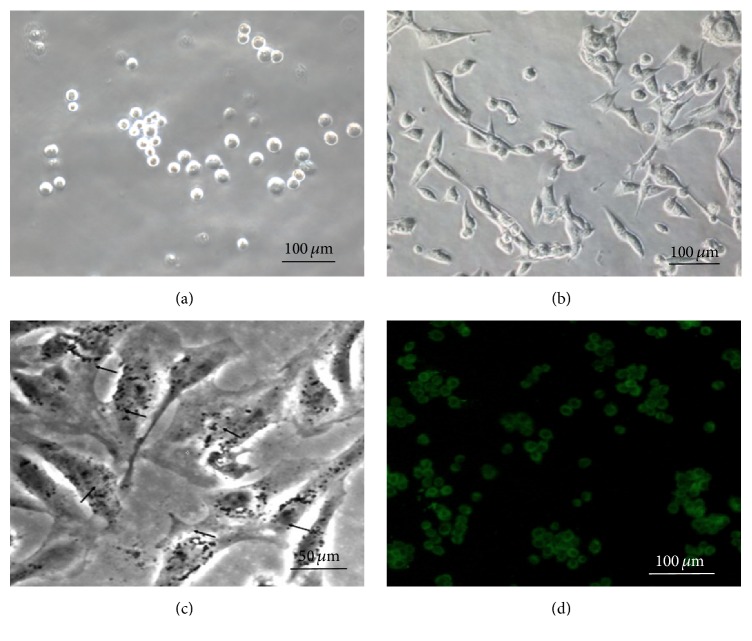
Kupffer cells isolation and identification. Kupffer cells were isolated from mouse liver; representative images of the morphology were shown at the initial (a) and after 12 h incubation (b). To further identify the Kupffer cells, the phagocytosis ability and marker were detected. Representative images of the ink engulfment (c) and F4/80 fluorescent staining (d) were shown.

Kupffer cells were isolated from the mouse liver, and the morphology was observed at the initial (Figure 1(a)) and after 12 h incubation (Figure 1(b)). The phagocytic activity of the cells was validated by engulfment of ink (Figure 1(c)), and the Kupffer cells were further identified by fluorescently labeled F4/80 antibody (Figure 1(d)).

### 3.2. Properties of SNN-Containing Serum

**Figure 2:**
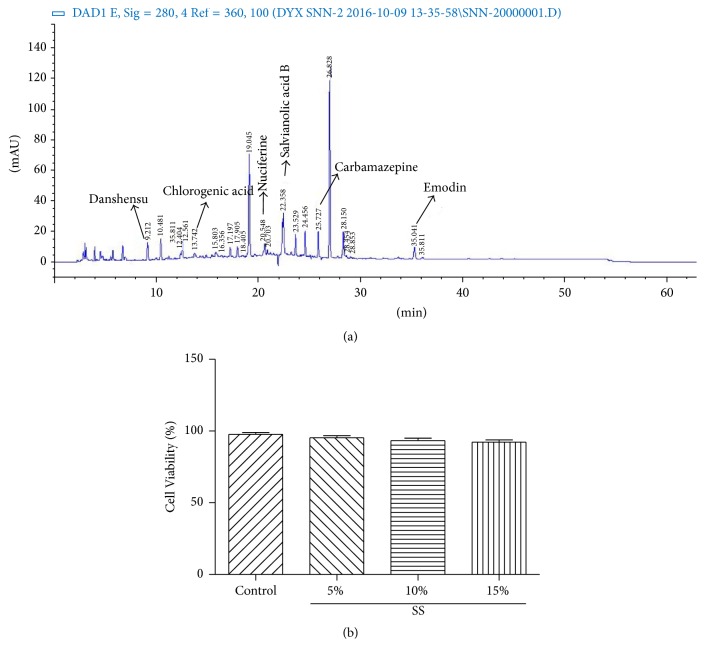
The properties of SNN-containing serum. Qualification of the main ingredients with HPLC (a). Different concentrations of SNN-containing serum (5%, 10%, 15%) on Kupffer cell viability (b). SS: SNN-containing serum. (b) is originated from GraphPad Prism 5.01, and data is linked.

SNN-containing sera collected from rats were qualified by HPLC according to Chinese Pharmacopoeia. Results indicated that the serum contained Danshen, Chlorogenic acid, nuciferine, Salvianolic acid, carbamazepine, and emodin (Figure 2(a)). Cell viability of isolated Kupffer cells did not show any difference upon SNN-containing serum treatment (5%, 10%, and 15%) (Figure 2(b)).

### 3.3. SNN-Containing Serum Reduced Inflammation of LPS Stressed Kupffer Cells

**Figure 3:**
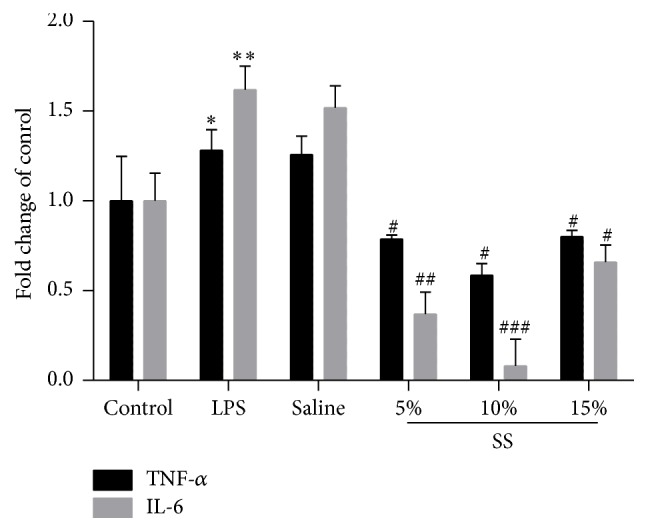
SNN-containing serum inhibits inflammation in LPS stressed Kupffer cells. LPS stressed Kupffer cells were treated with different concentrations of SNN-containing serum (5%, 10%, and 15%) or vehicle; TNF-*α* and IL-6 mRNA expression were detected. Experiments were repeated three times for each group, ^*∗*^*P* < 0.05 and ^*∗∗*^*P* < 0.01 versus control; ^#^*P* < 0.05, ^##^*P* < 0.01, and ^###^*P* < 0.001 versus LPS stressed cells. SS: SNN-containing serum. Origin from GraphPad Prism 5.01, and data is linked.

Coincubation Kupffer cells with LPS (1 *μ*g/ml) for 24 h induced significant increase of TNF-*α* and IL-6 mRNA expression; while saline serum did not change the inflammatory cytokines expression, the SNN-containing serum significantly decreased TNF-*α* and IL-6 mRNA expression, with 10% concentration prior to other concentrations (5% and 15%) in IL-6 expression decrease (Figure 3).

### 3.4. Microarray Analysis of Differentially Expressed miRNAs

**Figure 4:**
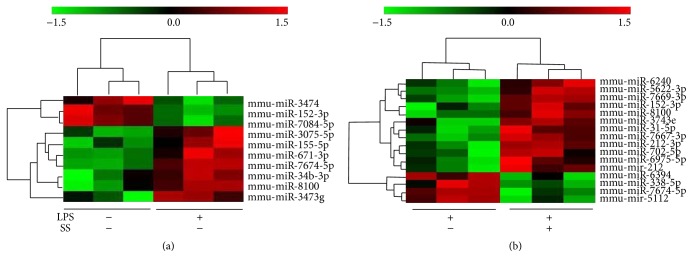
miRNA profiles in Kupffer cells under different conditions. (a) Comparison of observed miRNAs in normal Kupffer cells and LPS stressed Kupffer cells. (b) Comparison of observed miRNAs in LPS stressed (untreated) Kupffer cells and 10% SNN-containing serum treated cells. The columns and rows represent samples and particular miRNAs. Experiments were repeated three times for each group; SS: SNN-containing serum.

Different miRNAs expressed in Kupffer cells with conventional medium and with LPS addition were shown in Figure 4(a). 10 different miRNAs were identified between LPS stressed and unstressed Kupffer cells (Tables 2 and 3). We further analyzed the miRNAs alteration with SNN-containing serum treatment and demonstrate different miRNAs expressed (Figure 4(b)). 16 miRNAs responded to the SNN-containing serum treatment. Through comparing the differently expressed miRNAs among the three groups, miR-152-3p and miR-7674 were picked out to be the targets of SNN-containing serum. MiR-152-3p was significantly decreased (1.64 fold) in LPS stressed Kupffer cells, and SNN-containing serum restored its expression. miR-7674 was remarkably increased in LPS stressed Kupffer cells, and SNN-containing serum blocked its increase.

### 3.5. MiR-152-3p Mimics Inhibited Inflammation in LPS Stressed Raw.264.7 Cells

**Figure 5:**
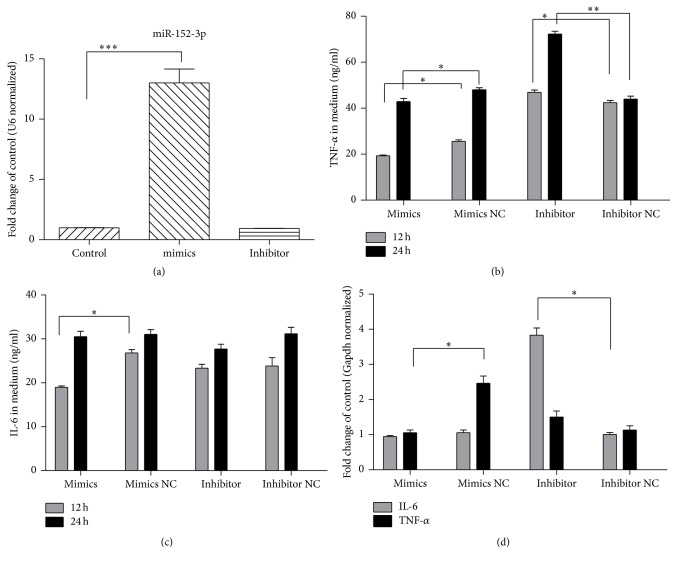
MiR-152-3p in Raw264.7 cells. (a) MiR-152-3p expression upon miR-152-3p mimics or inhibitors transfection. (b) TNF-*α* in the medium upon miR-152-3p mimics or inhibitor pretreatment. (c) IL-6 levels in medium upon miR-152-3p mimics or inhibitor pretreatment. (d) IL-6 and TNF-*α* levels mRNA expression upon miR-152-3p mimics or inhibitor pretreatment. Experiments were repeated three times for each group; ^*∗*^*P* < 0.05, ^*∗∗*^*P* < 0.01, and ^*∗∗∗*^*P* < 0.001 between two groups accordingly. Origin from GraphPad Prism 5.01, and data is linked.

To identify the effect of miR-152-3p on inflammation, we added miR-152-3p mimics and inhibitor to Raw.264.7 cells, respectively. In conventional incubated Raw.264.7 cells, miR-152-3p expression showed 12-fold increase with mimics compared to control cells (Figure 5(a)); however, the inhibitor fails to further decrease the miR-152-3p expression in Raw264.7 cells (Figure 5(a)).

In LPS stressed Raw264.7 cells, miR-152-3p mimics significantly decreased TNF-*α* level in the medium with both 12 h and 24 h treatment (Figure 5(b)), and IL-6 level in the medium significantly decreased with 24 h miR-152-3p mimics treatment (Figure 5(c)), indicating miR-152-3p increase had anti-inflammatory effect in LPS stressed Raw264.7 cells. In contrast, the miR-152-3p inhibitors, despite failing to further decrease miR-152 in conventionally incubated cells, promoted TNF-*α* secretion in LPS stressed Raw264.7 cells (Figure 5(d)).

### 3.6. MiR-152-3p Inhibitors Counteracted the Beneficial Effect of SNN-Containing Serum

**Figure 6:**
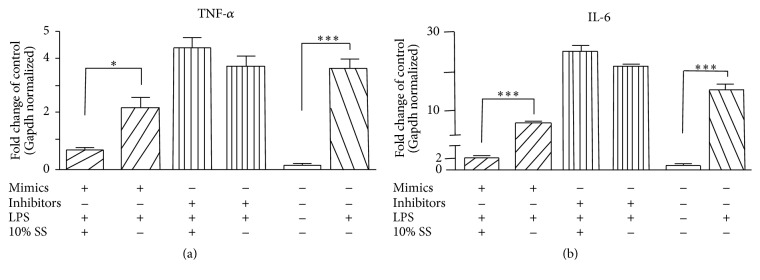
The combination effect of miR-152-3p and mimics (inhibitor) on TNF-*α* and IL-6 expression. (a) The change of TNF-*α* mRNA expression under various conditions. (b) The change of IL-6 mRNA expression under various conditions. ^*∗*^*P* < 0.05; ^*∗∗∗*^*P* < 0.001 between two groups accordingly.

We further assessed the combination effects of the SNN-containing serum with mimics or inhibitors in LPS stressed Raw264.7 cells. It revealed that both miR-152-3p mimics and combination of SNN-containing serum with mimics significantly decreased the mRNA expression of TNF-*α* and IL-6 in LPS stressed Raw264.7 cells (Figures 6(a) and 6(b)), and the combination showed priority compared to mimics alone, suggesting the SNN-containing serum could synergistically interact with the mimics. As expected, the miR-152-3p inhibitors both alone and in combination with SNN-containing serum failed to decrease the inflammatory cytokines; the TNF-*α* and IL-6 expression did not show any difference with untreated cells (Figures 6(a) and 6(b)), indicating that the inhibitors counteract the beneficial effect of SNN-containing serum.

## 4. Discussion

In the present study, we observed the anti-inflammatory effect of SNN-containing serum in LPS stressed Kupffer cells and comprehensively analyzed the miRNAs in Kupffer cells under different conditions. Through comparing the miRNA profiles, we reported for the first time that LPS stimulation decreases miR-152-3p levels in LPS stressed macrophages, and miR-152-3p levels were increased upon SNN-containing serum treatment. We identified miR-152-3p as the potential epigenetic regulator that underlined the pharmacological effect of SNN-containing serum. In addition, epigenetic regulator was verified in Raw264.7 macrophage cell line, and the regulation under the beneficial effect of SNN-containing is further confirmed.

Sensitization of Kupffer cells to gut-derived LPS was shown to contribute to the initiation and progression of NASH; Kupffer cells-derived TNF-*α* has been identified as an important mediator of steatosis, inflammation, and hepatocyte damage. NAFLD is the liver manifestation of the metabolic syndrome and the most common cause of chronic liver disease in developed countries [7]. In a proportion of the patients with simple steatosis, associated liver inflammation (NASH) develops, additionally characterized by hepatocyte ballooning degeneration, and such patients are prone to development of liver fibrosis [16]. The pathogenesis of NASH has traditionally been presented as a “two-hit” and “multihits” theories, with hepatocyte lipid accumulation as the first hit and inflammatory factors as the following hits. Macrophages are the main source of cytokines and macrophage-derived proinflammatory cytokines; TNF-*α* and IL-6 have been considered to contribute to the pathogenesis of NASH [17, 18]. Liver resident Kupffer cells, as first responders, are responsible in part for the recruitment of blood-derived monocytes upon liver injury. Both the activation of Kupffer cells and infiltration of blood-derived monocytes/macrophages have been shown to be essential for the perpetuation of liver inflammation [19]. Studies in rodents demonstrated that depletion of Kupffer cells by clodronate results in reduced liver injury, steatosis, and monocyte infiltration [20].

By using high-throughput technology of microarray chips, we successfully identified miR-152-3p and miR-7674 that altered in response to SNN-containing serum treatment. Since miR-7674 is a newly found miRNAs, we focused on studying the role of miR-152-3p. Previous studies showed that expression of miR-152-3p was significantly downregulated in the liver of* db/db* mice and mice fed a high fat diet, and inhibition of miR-152-3p induced impaired glycogenesis in hepatocytes [21]. PTEN is considered to be participating in the process [21]. In addition, miR-152-3p could inhibit apoptosis in the brain and act as a protective factor during hypoxia by repressing PTEN [22]. MiR-152 inhibits cell proliferation and colony formation in liver cancer stem cells [23] and plays a critical role in immune regulation and spontaneous tolerance induction in mouse liver transplantation [24]. Ectopic excess of miR-152 prevents migration of breast cancer cells, for miR-152 targeting DNMT1 mRNA and inhibits its protein expression [25].

Salvianolic acid B is one of the metabolites identified in our SNN-containing serum; coincidently, Salvianolic acid B has been reported to suppress the activation of Hepatic stellate cells (HSCs) in CCl4-treated mice and mouse primary HSCs, leading to decreased cell proliferation, type I collagen, and alpha-smooth muscle actin. The antifibrotic effects are related to the increase of miR-152, whereas miR-152 inhibitor could reverse Salvianolic acid B mediated effect [26] in Kupffer cells; these results are consistent with our data, suggesting miR-152 could be a potential drug target and promising strategy for NASH related diseases.

We have confirmed that SNN-containing serum inhibited inflammation via upregulating miR-152; whereas miR-152 inhibitor could block the benefit effect, combination of SNN-containing serum with miR-152 mimics could further enhance the anti-inflammatory effect, indicating they two were synergistically worked. However, since the formula is composed of multiple ingredients, we could not exclude the other interactions in the process. In addition, we also found the alteration of miR-7674; since it is a new member of the miRNA family, the functions related are still unknown and need further exploration.

## 5. Conclusion

Applying seropharmacological method, we have thoroughly analyzed the miRNAs profiles in Kupffer cells and identified miR-152 as the targeted SNN-containing serum in improving LPS induced inflammation; the epigenetic regulation was further verified in macrophages and confirmed that the anti-inflammatory effect of SNN-containing serum is via upregulating miR-152.

## Figures and Tables

**Table 1:** Sequences of the primers use for PCR.

Gene	Sense	Antisense
TNF-*α*	AGTGTGGGAAGCTGTCT	CAGCCTTAAGACAATTGGG
IL-6	ATGAAGTTCCTCTCTGCAAG	GTGTAATTAAGCCTCCGACT
U6	CTCGCTTCGGCAGCACA	AACGCTTCACGAATTTGCGT
GAPDH	GGTTGTCTCCTGCGACTTCA	GGTGGTCCAGGGTTTCTTACT

**Table 2:** Differentially expressed miRNAs in Kupffer cells with or without LPS stress.

miRNA	Fold change	*P* value	Regulation
mmu-miR-3075-5p	1.5853548	0.028243892	Up
mmu-miR-155-5p	2.7438154	0.03547192	Up
mmu-miR-671-3p	3.2789745	0.012668605	Up
mmu-miR-7674-5p	1.843111	0.002115972	Up
mmu-miR-34b-3p	2.4408915	0.049670372	Up
mmu-miR-8100	1.5702492	0.026956348	Up
mmu-miR-3473g	1.7498285	0.040682547	Up
mmu-miR-3474	1.5187571	0.023498446	Down
mmu-miR-152-3p	1.6380094	0.005590409	Down
mmu-miR-7084-5p	1.5028241	0.006869066	Down

**Table 3:** Differentially expressed miRNAs in LPS stressed Kupffer cells with or without SNN containing serum treatment.

miRNA	Fold change	*P* value	Regulation
mmu-miR-6240	1.7299925	0.024313163	Up
mmu-miR-5622-3p	2.009634	0.006489413	Up
mmu-miR-7669-3p	1.9773362	0.004524522	Up
mmu-miR-152-3p	1.7316191	0.02109938	Up
mmu-miR-8100	1.586356	0.007805437	Up
mmu-miR-3473e	1.6918215	0.027178766	Up
mmu-miR-31-5p	2.3238704	0.022005625	Up
mmu-miR-7667-3p	4.128067	0.003611936	Up
mmu-miR-212-3p	2.389236	0.0099163	Up
mmu-miR-702-5p	2.0031996	0.03293999	Up
mmu-miR-6975-5p	1.6072179	0.043497216	Up
mmu-miR-212	1.6010168	0.02455883	Up
mmu-miR-6394	1.5849638	0.02723776	Down
mmu-miR-338-5p	1.5117552	0.029625554	Down
mmu-miR-7674-5p	2.0667706	0.011290047	Down
mmu-miR-5112	1.5624359	0.013743355	Down
